# Hepatitis E Virus Genotype 3 in Wild Rats, United States

**DOI:** 10.3201/eid1808.120070

**Published:** 2012-08

**Authors:** Justin B. Lack, Kylie Volk, Ronald A. Van Den Bussche

**Affiliations:** Oklahoma State University, Stillwater, Oklahoma, USA

**Keywords:** hepatitis E virus, viruses, rats, zoonosis, genotype 3, viral RNA, PCR, United States

## Abstract

The role of rodents in the epidemiology of zoonotic hepatitis E virus (HEV) infection has been a subject of considerable debate. Seroprevalence studies suggest widespread HEV infection in commensal *Rattus* spp. rats, but experimental transmission has been largely unsuccessful and recovery of zoonotic genotype 3 HEV RNA from wild *Rattus* spp. rats has never been confirmed. We surveyed *R. rattus* and *R. norvegicus* rats from across the United States and several international populations by using a hemi-nested reverse transcription PCR approach. We isolated HEV RNA in liver tissues from 35 of 446 rats examined. All but 1 of these isolates was relegated to the zoonotic HEV genotype 3, and the remaining sequence represented the recently discovered rat genotype from the United States and Germany. HEV-positive rats were detected in urban and remote localities. Genetic analyses suggest all HEV genotype 3 isolates obtained from wild *Rattus* spp. rats were closely related.

## Abstract 2

Rodents infected with this virus may be a serious threat to public health.

Hepatitis E virus (HEV) is a major cause of acute hepatitis in developing countries, in which outbreaks arise most often through fecal contamination of drinking water or after flooding (*1*). Major outbreaks have been reported in India, Southeast Asia, Africa, and Mexico, and mortality rates are considerable (20%–30%) among pregnant women (*1*). In industrialized countries, HEV infections are reported sporadically and contamination of drinking water is an unlikely source, but cases are increasing as diagnostic tests are being performed more frequently (*2*). Moreover, zoonotic transmission of HEV through consumption of undercooked pork and deer meat has been confirmed (*3**,**4*), and detection of HEV in many mammalian hosts suggests the potential for multiple zoonotic sources of HEV infection in industrialized countries (*5*).

There are currently at least 4 genotypes of HEV known to infect humans. Genotypes 1 and 2 have been identified only from humans and are responsible for most outbreaks in developing countries (*6*). Genotypes 3 and 4 are believed to be involved in zoonotic transmission and have been isolated from swine (domesticated pig and wild boar), deer, mongoose, rabbits, cattle, and humans (*5*). Additional strains not known to infect humans have also been identified in rats and chickens, and the genetic diversity of HEV is only beginning to be understood.

Within the United States, HEV infections have been identified in travelers who have visited developing countries (*7*), and for several at-risk groups in the United States (i.e., swine veterinarians and farmers), the high number of reported seropositive persons is caused by swine–human contact (*8**,**9*). However, seroepidemiologic examinations of blood banks in the United States and other industrialized countries have shown high proportions of samples positive for antibodies against HEV (excluding persons who had traveled to HEV-endemic countries), but this finding was true in urban areas in which swine–human contact is absent (*8**,**10**,**11*).

HEV RNA has been detected in livers from commercially raised pigs (*12*) and represents an additional potential reservoir of infection. However, consumption of raw pork and wild game is uncommon in the United States, although it is a common practice in other industrialized nations in which high HEV seroprevalence has been reported (i.e., France) (*13*). This finding suggests that in addition to travel to HEV-endemic regions and swine–human contact, additional reservoirs of HEV infection exist in the United States, and evidence has accumulated indicating rodents as a potential HEV reservoir (*14**–**18*). In a survey of 26 rodent species in the United States, Favorov et al. (*14*) found 14 species of rodents seropositive for antibodies against HEV. Urban populations had ≈2× the proportion of seropositive rats relative to rural populations, and commensal *Rattus* spp. (*R. rattus* and *R. norvegicus*) rats had the highest proportion of seropositive animals (*14*).

The role of wild *Rattus* spp. rats as reservoirs in the epidemiology and transmission of HEV is unclear, but their ubiquity in urban environments and unparalleled propensity for carrying zoonotic pathogens makes them an obvious target of investigation. Multiple studies have reported finding IgG and IgM against HEV in *R. norvegicus* and *R. rattus* rat populations across the United States and Asia (*14**–**18*). Shukla et al. (*19*) successfully infected cell lines from *Mus musculus* mice, murid rodents closely related to *Rattus* spp. rats, with HEV genotype 3. In addition, Maneerat et al. (*20*) experimentally infected laboratory *R. norvegicus* rats with HEV isolated from infected humans, although the genotype of the infecting virus was unclear. After infection, the human virus strain effectively replicated in multiple tissues, and HEV RNA was detected in feces and serum for >30 days postexposure, suggesting that human strains of HEV can replicate in and be transmitted by *R. norvegicus* rats. However, recent discovery of a rat-specific strain of HEV not known to infect humans (*21**–**23*) suggests that high seroprevalence of antibodies against HEV may be caused by cross-reactivity rather than widespread infection with a human-infecting HEV genotype.

We used a reverse transcription PCR (RT-PCR) approach to survey *R. rattus* and *R. norvegicus* rats for HEV RNA. Our analysis detected HEV RNA in liver tissues from *R. rattus* and *R. norvegicus* rats at many localities across the United States. Sequencing of DNA from RT-PCR–positive samples indicated widespread infection with zoonotic HEV genotype 3: one rat in California was positive for the rat-specific strain. These findings suggest that wild *Rattus* spp. rats are competent hosts for genotype 3 HEV.

## Materials and Methods

### Rat Tissues

We obtained liver tissue samples from 446 *R. rattus* and *R. norvegicus* rats from museum collections (Technical Appendix) covering localities primarily in the United States (15 states) plus additional samples from China, Honduras, Madagascar, Mexico, Nicaragua, Peru, Russia, and Vietnam (Table). To maximize the likelihood of intact viral RNA, all liver samples selected were dissected from recently euthanized animals, immediately frozen, and maintained at −80°C until thawed for extraction.

**Table:** *Rattus* spp. rats tested for hepatitis E virus RNA*

Location	Species and sample size		
	*R. norvegicus*	*R. rattus*	No. positive
United States			
Aleutian Islands, Alaska	18	7	6
San Francisco Bay Area, California	19	112	12
Gainesville, Florida	NA	21	4
Oklahoma City, Oklahoma	1	NA	1
Memphis, Tennessee	16	NA	6
San Angelo, Texas	2	11	2
Little Rock, Arkansas	2	6	0
San Diego, California	8	5	3
Panama City, Florida	NA	24	0
Key Largo, Florida	NA	5	0
Spencer, Indiana	NA	10	0
Baton Rouge, Louisiana	NA	12	0
Prentiss, Mississippi	NA	1	0
Bernalillo, New Mexico	2	NA	0
Union County, Pennsylvania	40	NA	1
Corvallis, Oregon	4	NA	0
Houston, Texas	NA	8	0
Austin, Texas	NA	14	0
Kerns, West Virginia	1	NA	0
Seattle, Washington	1	5	0
Vietnam	NA	18	0
China	NA	5	0
Honduras	NA	2	0
Madagascar	NA	5	0
Mexico	1	2	0
Nicaragua	1	11	0
Peru	NA	16	0
Russia	30	NA	0

*NA, no samples were available.

### Hemi-nested RT-PCR

Total RNA was extracted from ≈30 mg of liver tissue by using the RNeasy Mini Kit (QIAGEN, Valencia, CA, USA). We used a modification of the broad-spectrum RT-PCR approach of Johne et al. (*22*) to amplify a 334-bp fragment of HEV open reading frame 1 (ORF1). Primers were selected for their ability to amplify ORF1 from all known HEV genotypes, and all primer sequences are reported by Johne et al. (*22*). Attempts to amplify the ORF1 fragment from total extracted RNA resulted in amplification of a portion of an unidentified transcript in all *R. rattus* rat samples. When we sequenced the amplicon, it was clear that spurious amplification was caused by nonspecific binding of primer HEV-cas. To circumvent this problem, we used a hemi-nested approach, with the initial RT-PCR using the HEV-cs/HEV-casN primer combination and the nested PCR using the HEV-csN/HEV-casN primer combination. With the exception of the change in primer combinations, all other aspects of amplification followed the protocol of Johne et al. (*22*). Positive PCR amplicons (verified by agarose gel electrophoresis) were purified by using the Wizard SV Gel PCR Clean-Up System (Promega, Madison, WI, USA) and sequenced in both directions by using nested PCR primers (HEVcsN/HEVcasN).

Given the high sensitivity of a nested PCR approach, contamination can be a major issue and has been cited as problematic in investigations of HEV in rodents (*24*). Exceptional effort was made to ensure that no contamination occurred. All PCR steps were conducted in a sterile environment, under a laminar flow hood, and all surfaces, tubes, and equipment were UV irradiated between each PCR. This study was conducted in a newly constructed laboratory in which no HEV samples (or any other animal samples) had been handled, extractions were conducted in a room separate from that used for PCR amplifications, and all steps (extraction, RT-PCR, and nested PCR) included negative controls. In addition, a single HEV genotype 3 isolate was used as a positive control in PCRs, and we sequenced this isolate for the same locus targeted for the *Rattus* spp. rat samples. Any *Rattus* spp. rat HEV isolate exhibiting 100% nt identity to this positive control sequence was excluded as a contaminant.

### Phylogenetic Analyses

**Figure A1:**
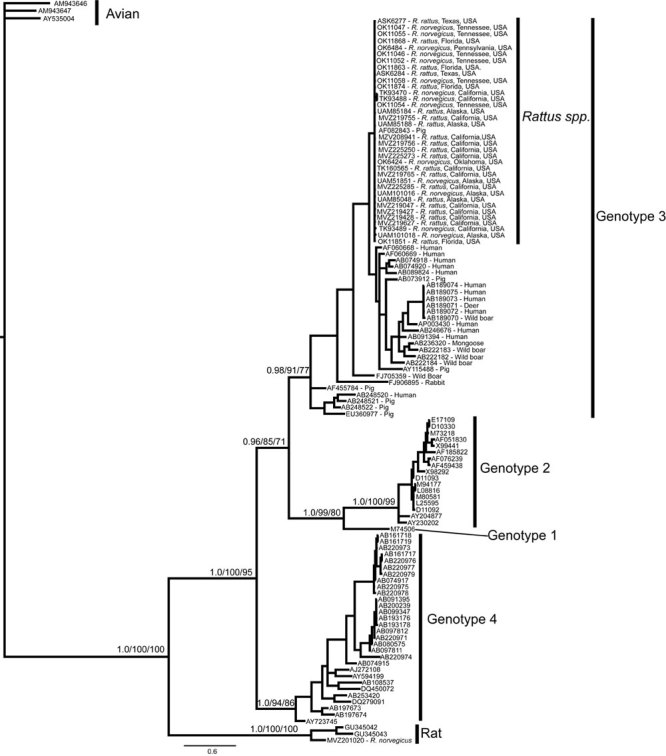
Bayesian phylogram resulting from analysis of a 334-bp fragment of hepatitis E virus (HEV) open reading frame 1. Node labels represent Bayesian posterior probabilities/maximum-likelihood bootstrap/maximum-parsimony bootstrap values, respectively, and are given only for nodes at the base of each genotype and nodes uniting the genotypes. For sequences obtained in this study from isolates from wild rats collected in the United States, terminal taxa labels correspond to tissue accession numbers shown in the Technical Appendix, followed by the species from which the tissue originated and the source population. For all other HEV genotype 3 sequences, the GenBank accession number is given, followed by the species from which the viral RNA was isolated. For all other sequences included, only the GenBank accession number is given. Scale bar indicates nucleotide substitutions per site.

In addition to the sequences we generated, we downloaded all complete HEV genome sequences from GenBank (accession numbers are shown in Figure A1) and extracted the ≈334-bp homologous portion of ORF1 from each genome. Total sequences were aligned by using the MAFFT aligner (*25*) implemented in Geneious version 5.5 (*26*). We conducted Bayesian, maximum-parsimony, and maximum-likelihood phylogenetic analyses on the combined alignment by using the avian HEV strain as an outgroup. For Bayesian analysis conducted in MrBayes version 3.2 (*27*), we partitioned the alignment by codon position and used a generalized time reversible + invariant sites + Γ substitution model, which Modeltest version 3.7 (*28*) indicated to be most appropriate. The analysis was run for 15,000,000 generations sampled every 1,000 generations, and burn-in values were determined empirically by evaluating likelihood scores. For maximum-parsimony analysis, we used tree bisection/reconnection branch swapping, 25 random additions of input taxa, and 1,000 bootstrap replicates to assess node support. For maximum-likelihood analysis, we used a generalized time reversible + invariant sites + Γ substitution model as indicated above, nearest-neighbor interchange branch-swapping, and 500 bootstrap replicates to assess node support. We generated a haplotype network for sequences generated in this study by using TCS software (*29*).

## Results

We excluded 7 isolates sequenced from 1 PCR batch that matched the positive control sequence. No subsequent matches with the positive control were detected, and no contamination was detected in negative controls. We identified 35 (7.85%) *Rattus* spp. rats positive for HEV by PCR from 446 rats examined. Most positive samples were from California (15 rats), but some were from rats in Tennessee, Florida, Oklahoma, Pennsylvania, Texas, and Alaska (Table). Phylogenetic analysis placed 34 of these positive rat samples in a closely related group within the HEV genotype 3 clade (Figure A1) termed subclade 3a by Lu et al. (*6*). This placement was supported in all analyses. Mean pairwise uncorrected genetic distances between HEV genotype 3 sequences and other known HEV genotypes were 36.19%, 24.12%, 24.91%, 24.05%, and 33.52% compared with the avian genotype, genotype 2, genotype 1, genotype 4, and rat genotype, respectively. Network analysis showed that HEV genotype 3 sequences from *Rattus* spp. rats formed a tight cluster (Figure), differed by only a few mutations, and represented a single strain. Mean pairwise sequence divergence within *Rattus* spp. rat HEV genotype 3 sequences was 0.51%. A single sequence (AF082843) isolated from an HEV-infected pig was also in this group.

The single sequence not nested within the genotype-3 clade was isolated from an *R. norvegicus* rat from the San Francisco Bay area of California. Phylogenetic analyses placed it in a strongly supported clade with 2 other sequences isolated from *R. norvegicus* rats in Germany (Figure A1). Uncorrected genetic distances indicated that the California rat HEV sequence is ≈2× as divergent from the 2 sequences isolated in Germany (California vs. GU345042 = 13.98%; California vs. GU345043 = 14.86%) as the 2 Germany sequences are from each other (GU345042 vs. GU345043 = 7.78%). These findings suggest a degree of distinction between rat HEV strains from the United States and Europe.

## Discussion

A major conflict has surrounded the role of *Rattus* spp. rats (and other rodents) in HEV epidemiology since seroprevalence studies in the 1990s identified many species of rats positive for HEV antibodies in the United States and Asia (*15**–**18*). Maneerat et al. (*20*) infected 3 Wistar laboratory rats (*R. norvegicus*) with HEV (viral RNA was detected intermittently in feces for <30 days), but which genotype was used is unclear, and this result has not been duplicated (*9*). In addition, He et al. (*24*) isolated HEV genotype 1 from *R. rattus* and *Bandicota bengalensis* rats, but the study was later retracted because the authors were unable to rule out contamination as a source of detected viral RNA (*30*). More recently, Shukla et al. (*19*) successfully infected multiple *M. musculus* mouse cell cultures (in addition to infecting cow, rabbit, cat, dog, and chicken cultures) with HEV genotype 3, supporting the hypothesis that rodents may be competent hosts. However, there was substantial variation among different strains of HEV genotype 3 in the ability to infect cells derived from different hosts, including swine and human.

A recent attempt to infect adult Sprague-Dawley laboratory rats (*R. norvegicus*) with HEV genotypes 1, 2, and 3 failed (*23*). In this same study, infection of laboratory rats with the divergent rat genotype had limited success; only 25% of intravenously infected Sprague-Dawley rats and only 15.8% of nude rats seroconverted. This result is unexpected given that ≈80% of wild *R. norvegicus* rats from Los Angeles, California, where the study was conducted, were positive for IgG or IgM against HEV, suggesting that infection occurs in the wild (*23*). Johne et al. (*22*) also were unsuccessful in infecting rat liver cell lines with rat genotype HEV isolated from wild *R. norvegicus* rats from Germany. In contrast, we provide evidence of HEV genotype 3 infection in wild *R. rattus* and *R. norvegicus* rats.

Spread of HEV-positive rats indicates that infection in wild *Rattus* spp. rats is not restricted to any area of the United States or to urban areas. Our positive samples included both *Rattus* species of rat tested, and included the relatively remote Aleutian Islands in Alaska, and the urban San Francisco Bay area in California. Given the commensal nature of wild *Rattus* spp. rats and their ability to use human transportation vectors (i.e., commercial shipping) in dispersal, the prevalence of HEV in remote populations is not surprising. Recent work examining the genetic structure of *R. rattus* rats has shown that 2 mtDNA haplotypes have rapidly spread from their origin in India to every continent except Antarctica (*31**,**32*). Given the presence of HEV in domesticated animals (i.e., pigs) and human commensals (i.e., wild *Rattus* spp. rats), widespread domestication has likely enabled HEV to spread worldwide, potentially through interactions between humans, domesticated animals, and commensal rats. Furthermore, because *R. rattus* and *R. norvegicus* rats are sympatric over their contemporary range, lack of genetic distinction between strains infecting these 2 species is not unexpected (Figure A1, Figure).

In terms of infection rates, variation in handling of tissues from field-collected animals should be considered. Although we attempted to limit our analysis to the most well-preserved tissues, there is considerable variation among collection protocols and collectors in length of time between euthanasia and dissection, time between dissection and freezing, number of times tissues were thawed and frozen (i.e., in sorting, subsampling, shipping), and consistency of storage temperature. These factors can lead to nucleic acid degradation and negatively affect the ability to detect viral RNA. Therefore, our infection rate is likely not indicative of HEV infection rates in wild *Rattus* spp. rat populations.

Recent studies have reported major variation in diversity of competent mammalian hosts for various strains of HEV genotype 3 (*19*). Although seroprevalence studies have suggested infection rates ≤80% for HEV in US *Rattus* spp. rat populations (*15**,**23*), attempts to infect different laboratory strains of *R. norvegicus* rats with a genotype isolated from wild *R. norvegicus* rats have shown limited success; most attempts also failed in immunocompromised nude rats (*22**,**23*). These patterns, and the low genetic diversity of HEV-positive samples detected in this study (Figure), suggest that only a limited number of HEV genotype 3 strains may be capable of infecting *Rattus* spp. rats and other rodents (i.e., *Mus* spp.), possibly because of an HEV genotype 3/rat genotype recombinant.

Reduced genetic diversity of ORF1 sequences obtained from *Rattus* spp. rats requires further study, including sequencing genomes of these isolates to identify sequence diversity at other loci. Difficulty in transmitting virus from infected wild *R. norvegicus* rats into laboratory strains also indicates that certain life history or genetic characteristics may be essential for infection. Purcell et al. (*23*) reported a positive correlation between antibody prevalence and animal age in their study of seroprevalence of HEV in *Rattus* spp. rats, suggested that rats are readily infected in the wild, and that infection occurred in juvenile rats. This pattern is consistent with HEV infection in humans and swine (*33**,**34*), and suggests that infections should be attempted in wild and laboratory juvenile rats. Lending further support to this suggestion, the only report of major long-term infection (>30 days) of rats with HEV used weanling rats (*20*), and all other attempts we are aware of have used only adult rats (*23*). In addition, extreme variation in host specificity that Shukla et al. (*19*) observed among different HEV genotype 3 strains indicates the need for future transmission studies to include as many strains as possible.

Technical Appendix Rattus species rats examined for hepatitis E virus.

## Figures and Tables

**Figure:**
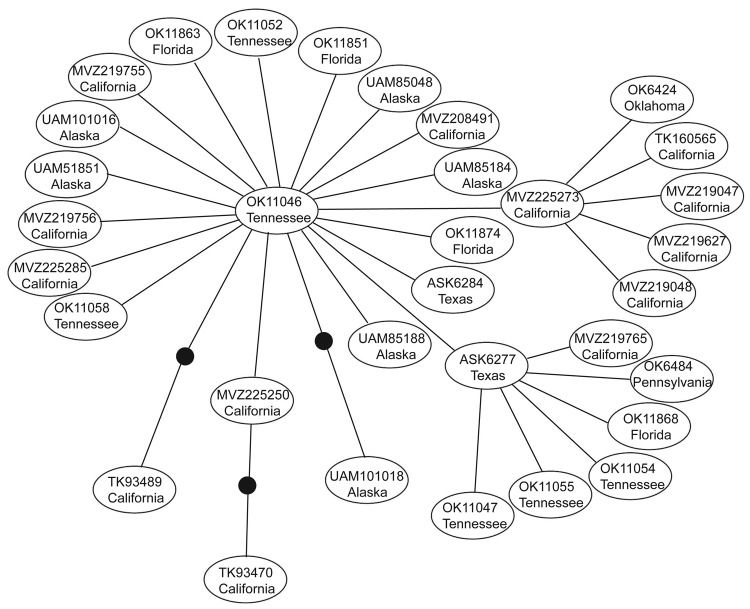
Genetic network showing the relationship among all hepatitis E virus genotype 3 sequences obtained in this study from isolates from wild rats collected in the United States. Each line represents a single mutational event and closed circles represent extinct or unsampled sequences. Each oval represents a single isolate, and the label corresponds to the tissue number shown in the Technical Appendix and the general sampling locality.
